# Case report: The stroma-rich variant of Castleman’s disease of hyaline-vascular type with atypical stromal cell proliferation and malignant potential: An exceptional rare case occurred in mediastinal lymph node

**DOI:** 10.3389/fonc.2023.1008587

**Published:** 2023-03-16

**Authors:** Xiaoxin Shi, Mengying Liao, Xiaomin Yin, Yaoli Chen, Chuqiang Huang, Weihua Yin, Jian Li

**Affiliations:** ^1^ Department of Pathology, Peking University Shenzhen Hospital, Shenzhen, China; ^2^ State Key Laboratory of Chemical Oncogenomics, Peking University Shenzhen Graduate School, Shenzhen, China

**Keywords:** Castleman’s disease, hyaline-vascular, malignant, stromal hyperplasia, p53

## Abstract

The stroma-rich variant of Castleman disease of hyaline-vascular type (SR-HVCD) is characterized by interfollicular proliferation of the fibroblastic, myofibroblastic, and/or histiocytic-derived stromal cells, occurred in a background of Castleman disease of hyaline-vascular type (HVCD). It has been considered as a hyperplastic disorder by far. Herein, we presented a case of a 40-year-old male suffering from an occupation in the right middle mediastinum. Microscopically, the lesion was characterized by atretic lymphoid follicles and overgrowth of the interfollicular spindle-shaped cells. Those spindle cells were histologically bland in some areas, while exhibited notable cellular atypia and focal necrosis in other areas. SMA and CD68 were immunostained with a subset of the spindle cells in both areas, whereas p53 staining was only perceived in areas with markedly cellular atypia. In addition, indolent T-lymphoblastic proliferation (iT-LBP) was present inside the lesion. The patient developed multiple sites metastases 4 months after surgery, and succumbed to the disease at 7 months. Our case demonstrates for the first time that SR-HVCD have a tumorigenesis potential rather than a simple hyperplastic process. Such disorder should be carefully evaluated to avoid underdiagnosis.

## Introduction

Castleman disease (CD) is an uncommon lymphoproliferative disorder and the incidence is estimated to be around 21 to 25 cases per million person-years (1, 2). According to the latest consensus of the Castleman Disease Collaborative Network (CDCN), CD encompasses a spectrum of conditions with heterogeneous etiologies, clinical manifestations, and histological characteristics (3). Clinically, it is grouped into unicentricity and multicentricity. Histologically, CD is classified into variants of hyaline-vascular (HVCD), plasma-cell, mixed type, hypervascular, and plasmablastic phenotype (3, 4).

HVCD accounts for nearly 90% of unicentric CD (2) and often manifested as a unicentric leison. The mediastinal lymph nodes is one of the most common sites of HVCD. Microscopically, HVCD is characterized by atretic follicles with hyalinized penetrating vessels and concentric onion-skin-like mantle zone. The interfollicular region generally exhibits proliferated vasculatures and unapparent lymphosinuses. In 1993, Danon et al. defined a new variant of HVCD, named as stroma-rich variant of Castleman disease of hyaline-vascular type (SR-HVCD) (5). Unlike the classical HVCD, SR-HVCD demonstrates a florid hyperplasia of actin-positive fibroblastic cells and KP1-positive histiocytic cells in the interfollicular zone. By definition, the area of interfollicular zone is required to be larger than that of follicular zone. Since then, a few studies further explored the clinicopathological features of this rare disorder (5–12). However, the etiology of SR-HVCD is still kept unknown. It is generally presumed to be a hyperplastic lesion based on the fact that all the reported cases exhibit a benign process and no associated malignancies have been recorded by far.

Interestingly, very few malignancies have been shown to be concurrently occurred with HVCD. One of them is follicular dendritic cell sarcoma (FDCS). The association between HVCD and FDCS was first unraveled by Chan et al. in 1994 (13), and was subsequently described by other researchers (13–15). Lymphoma, including Hodgkin’s disease and non-Hodgkin’s lymphoma, is another type of malignant tumors occasionally coexisted with HVCD (4, 16, 17). Herein, we presented an unusual case of SR-HVCD that exhibited a malignant profile, featured by dysplastic proliferation of the stromal cells and subsequent metastasis. The lesion’s morphologic features and related differential diagnosis were discussed in detail.

## Case presentation

**Figure 1:**
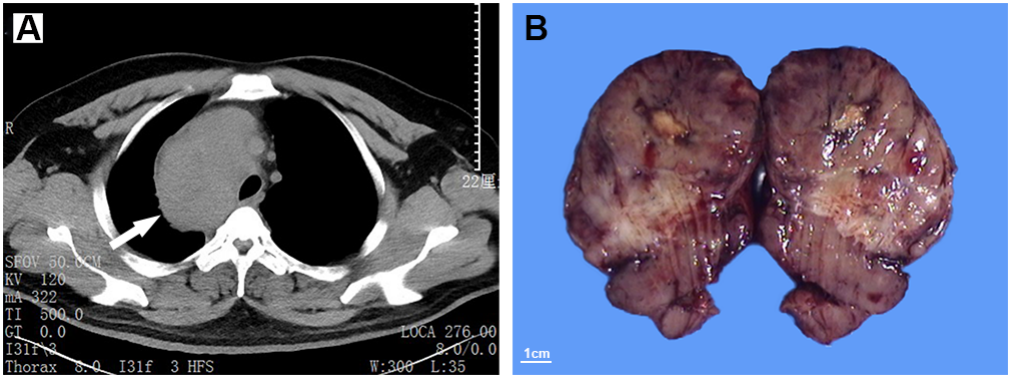
Computed tomography (CT) scans show an occupation in the right middle mediastinum (white arrow), with a CT value of 20-80 Hu **(A)**. The resected mass is well-demarcated, and exhibits a solid and lobulated appearance in cut surface **(B)**.

The patient was a 40-year-old male with a complaint of shortness of breath after activity. The symptom had lasted for around one month. No remarkable medical history has been stated by the patient. Computed tomography scans revealed a large mass in the right middle mediastinum. The maximum diameter was about 10cm (**Figure 1A**). Tracheal and the superior vena cava were found to be compressed. The laboratory findings (**Table 1**) showed a slight increase in soluble interleukin-2 receptor (SIL-2R) and lactate dehydrogenase (LDH). C-reactive protein (CRP), aspartate transaminase (AST), alanine transaminase (ALT), and creatinine did not show obvious abnormalites. The mass was then completely removed by surgery. Operative findings revealed the mass compressed the right upper lobe bronchus and the superior vena cava.

**Table 1:** The laboratory findings of the patient.

Laboratory findings (range)	Preoperation	At the fourth month after surgery
Hemoglobin (130-175g/L)	157	86
Red blood cell (4.3-5.8E+12/L)	5.29	3.1
White blood cell (3.5-9.5E+9/L)	7.30	17.89
Platelet (125-350E+9/L)	222	84
Aspartate transaminase (13-35U/L)	30.83	127.68
Alanine transaminase (0-50U/L)	29.75	83.43
Alkaline phosphatase (35-135U/L)	97.04	298.11
Total Bilirubin (0-26umol/L)	8.7	96
Blood urea nitrogen (2.60-8.80mmol/L)	4.79	5.28
Creatinine (41-81umol/L)	78.54	36.67
C-reactive protein (0-10mg/L)	10.60	37.69
Interleukin-6 (0-7.0pg/mL)	21.97	46.64
CA125 (0-35U/mL)	26.51	75.9
Soluble interleukin-2 receptor (180-265U/mL)	377.6	537.38
Lactate dehydrogenase (120-250U/L)	289.83	470.37

## Pathological features

**Figure 2:**
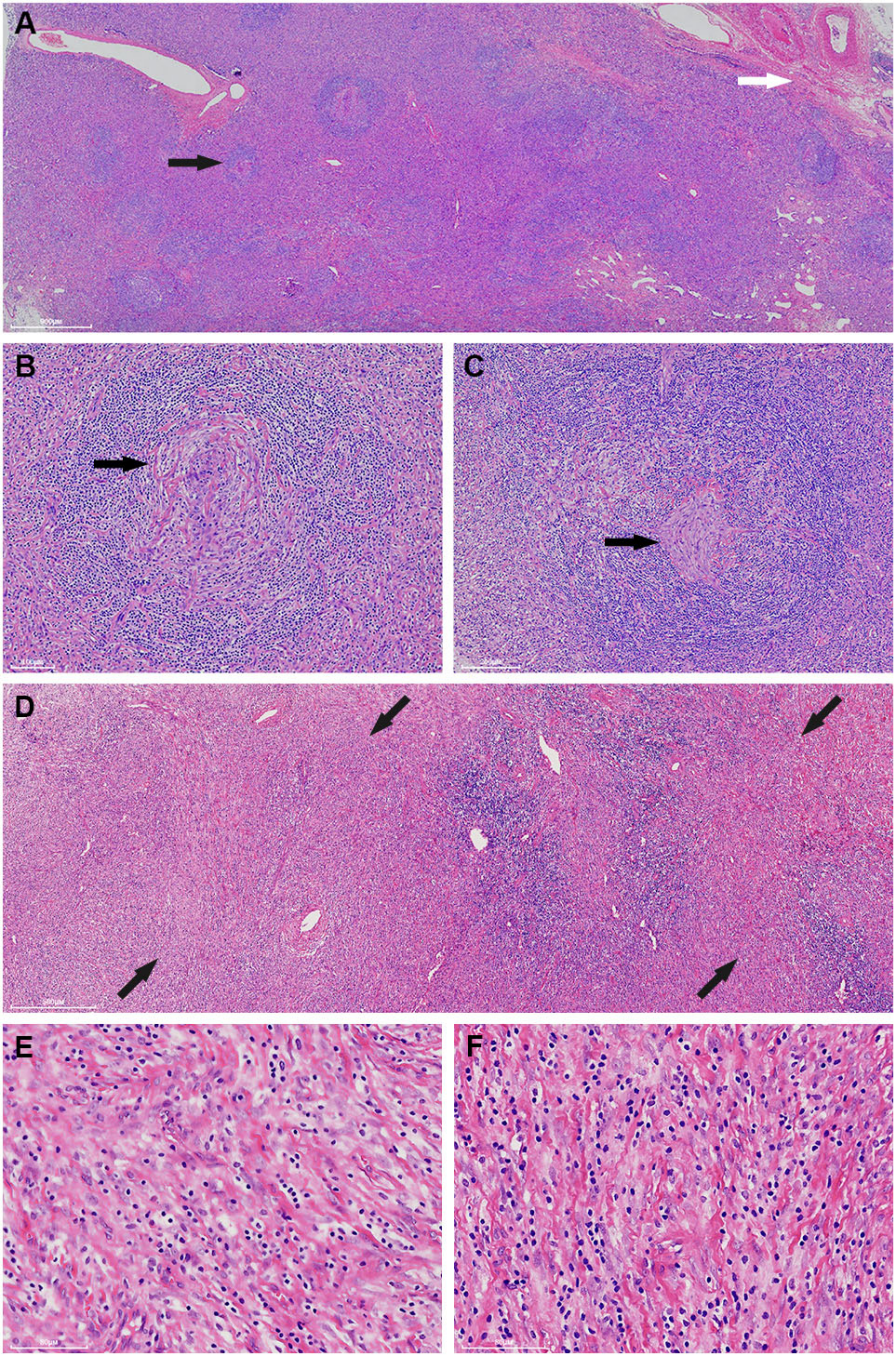
The mass generally exhibits a histologic structure of Castleman disease of hyaline-vascular type (HVCD). Beneath the capsule (white arrow), the lymphoid follicles are unevenly distributed and the follicles are generally atrophied (black arrow) **(A)**. The germinal centers are characterized by a depletion of lymphoid cells and proliferation of follicular dendritic cells (black arrow). The mantle zone lymphocytes are concentrically arranged and impart an onion-skin-like appearance **(B, C)**. Toward the central region, the interfollicular areas are widened by storiform-arranged spindle cells under low power (back arrow) **(D)**. Those spindle cells illustrate long, slender cytoplasm and vesicular to fine chromatin nuclei. No distinctive nuclear atypia is perceived in these fields **(E, F)**.

**Figure 3:**
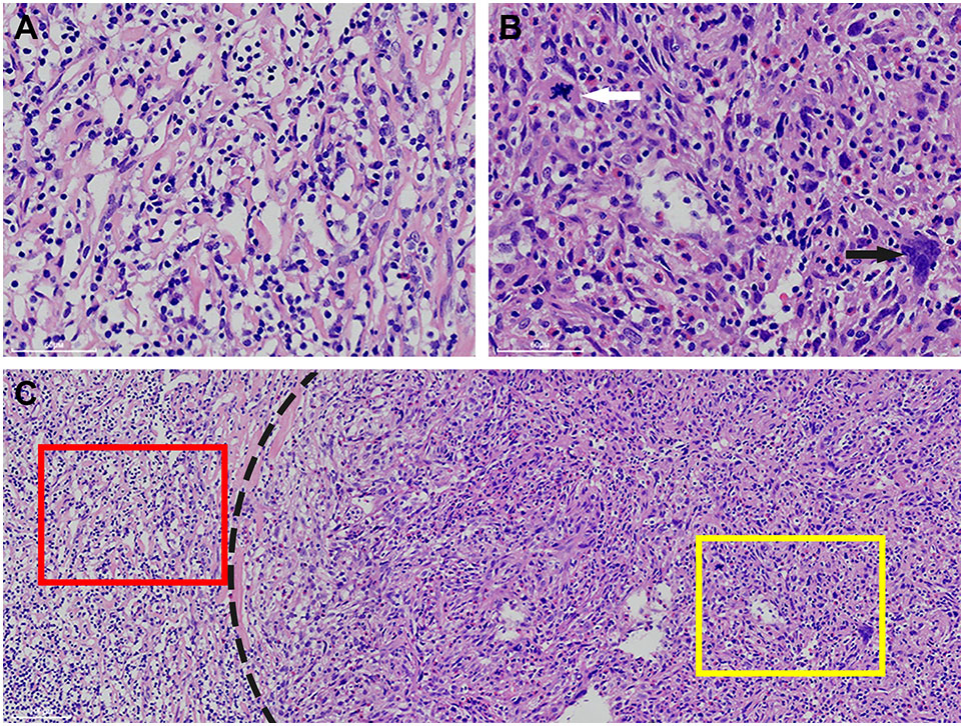
Apart from the cells with a bland appearance **(A)**, some of the spindle cells demonstrate remarkably pleomorphism. Abnormal mitoses (white arrow) and multinucleated tumor cells (black arrow) could be observed **(B)**. There exists a morphological transition between the bland (left side of the dot line) and atypical cellular regions (right side of the dot line). The high power fields of the red square and the yellow square are corresponding to **(A, B)**, respectively **(C)**.

Macroscopically, the mass was 10.5cm×8.7cm×7cm in size and grey to red in color. It was solid and nodular in cut surface and tenacious to soft in texture (**Figure 1B**). A fibrous capsule was observed in the periphery regions of the mass. Microscopically, the lymphoid follicles were unevenly distributed under the capsule (**Figure 2A**). Most of them were atretic in appearance. The hyperplastic follicular dendritic cells in germinal center and concentrically-arrayed lymphocytes in mantle zone could be easily observed (**Figures 2B, C**). Occasionally, penetrated sclerotic blood vessels were found. Toward to the central region, the lymphoid follicles were markedly attenuated. The interfollicular fields were dominated by the compact plump spindle cells with a fascicular and storiform arrangement (**Figure 2D**). In some areas, the spindle cells demonstrated slender cytoplasm and indistinct cellular borders. The nuclei were oval and/or elongated, with vesicular to fine chromatin and small nucleoli. No obvious nuclear atypia or pleomorphism was presented (**Figures 2E, F**, **3A**). By contrast, in other areas they exhibited enlarged and hyperchromatic nuclei, increased mitotic activity (5 per 10 high power fields), and atypical mitoses (**Figure 3B**, **Supplementary Figure 1**). Neoplastic necrosis was observed focally (**Supplementary Figure 2**). The morphologic transition could be perceived between the mild and dysplastic cellular areas (**Figure 3C**, **Supplementary Figure 1**). In addition, proliferated small vessels were found tightly intermixed with the stromal cells (**Supplementary Figure 3**). Moreover, small lymphocyte infiltrates were also noticed inside the lesion and distributed in a varied density. Those lymphocytes illustrated oval nuclei, darkly-stained chromatin and inconspicuous nucleoli (**Figures 2E, F**).

**Figure 4:**
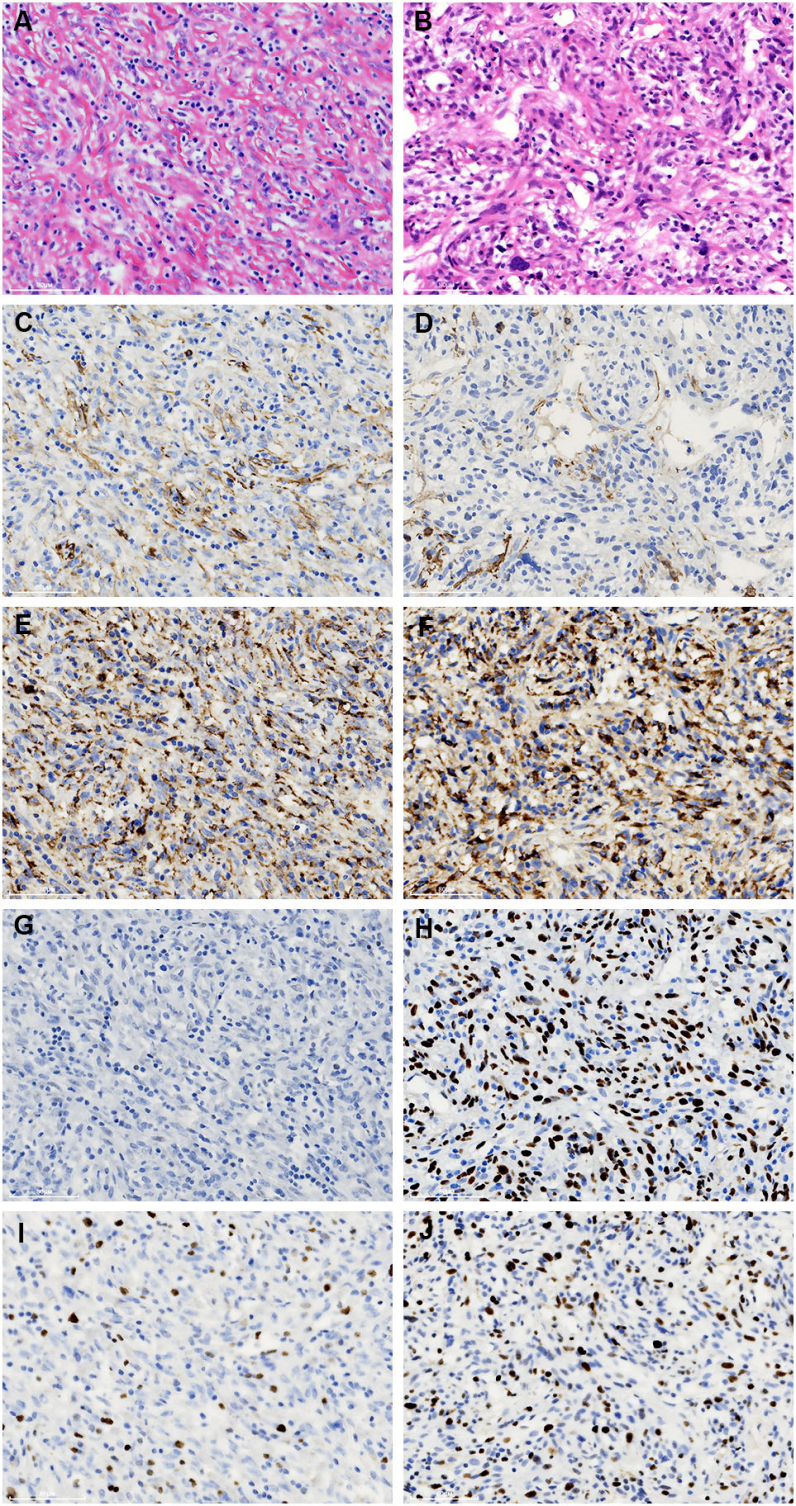
Comparison of the immunostaining features of the bland **(A, C, E, G, I)** and atypical spindle cells **(B, D, F, H, J)**. They are focally immunoreactive with SMA **(C, D)** and CD68 **(E, F)**. However, the staining of p53 is negative in the former **(G)**, while strongly expressed in the latter **(H)**. In addition, an increased ki-67 index is noticed in the atypical spindle cells **(I, J)**.

**Figure 5:**
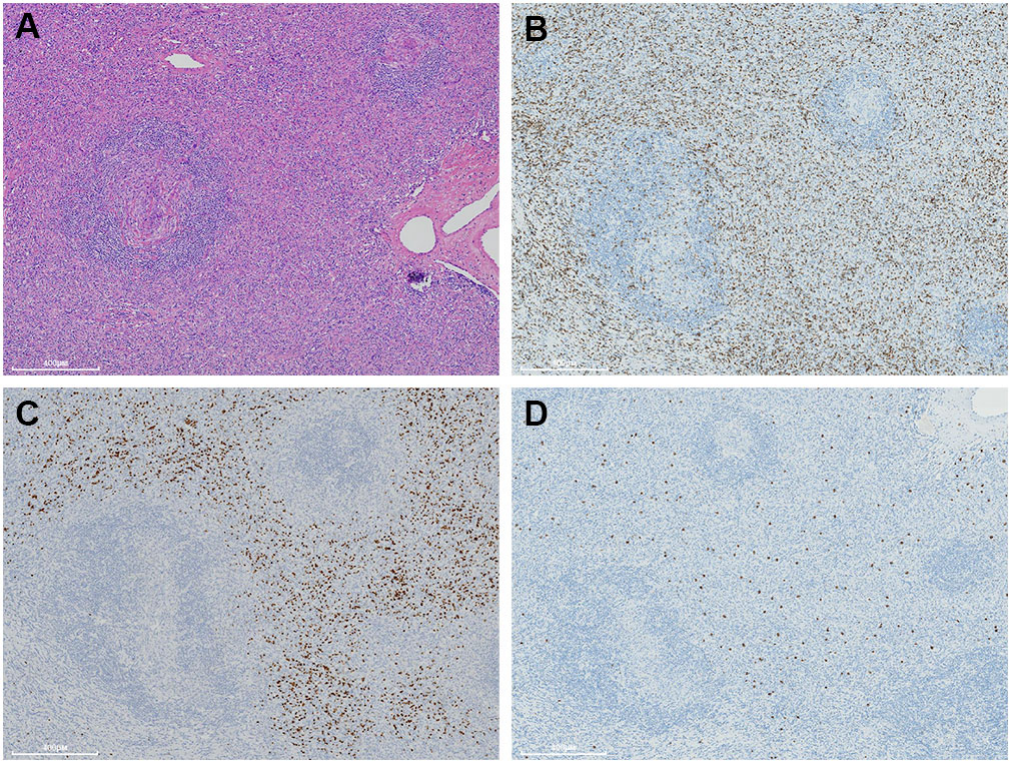
A moderate number of small lymphocytes are unevenly infiltrated inside the interfollicular regions **(A)**. They are variably immunostained with CD3 **(B)**, CD10 **(C)**, and TdT **(D)**.

Immunohistochemically, the spindle cells were focally stained with SMA and CD68 (**Figures 4A–F**). Strong P53 staining and increased Ki-67 index (20%) was detected in regions with notably cellular atypia, while negative P53 staining and low Ki-67 index (10%) was presented in spindle cells with benign morphology (**Figures 4G–J**). On the other hand, they were uniformly negative for CD21, CD23, CD35, SSTR2, D2-40, and CXCL13 (**Supplementary Figure 4**). Moreover, they also exhibited negative staining for CK-pan, CK5/6, CK8/18, CK19, P63, CD5, CD117, STAT-6, CD34, CD31, ERG, ALK, S-100, MDM2, CDK4, p16, TLE1, GLUT1, TRK, Muc4, and HHV-8. EBER *in situ* hybridization was negative either. FISH analysis by break-apart probes showed negative results for the rearrangements of EWSR1 and SS18 gene. The staining of CD20 highlighted B-cells in follicles. The interfollicular small lymphocytes were immunoreactive with CD3 and CD5, and some of them were positive for TdT and CD10 (**Figures 5A–D**). PCR analysis revealed there was no clonal rearrangement in T-cell receptor (TCR) beta and gamma gene.

## Post-surgery treatment and prognosis

After the pathological diagnosis was drawn, the patient received additional physical examination and no definite symptoms correlated with HVCD, such as myasthenia gravis, erythra, paraneoplastic pemphigus, and bronchiolitis obliterans were discerned. He received combined treatments of epidoxorubicin and bevacizumab after surgery and kept well for 3 months. Then he developed chest pain and weakness of lower limbs at 4 month. Magnetic resonance imaging revealed multifocal bone and liver metastasis (**Supplementary Figure 5**). The level of SIL-2R, LDH, AST, ALT, total bilirubin, CRP, CA125, and interleukin-6 were variably increased (**Table 1**). The patient refused biopsy on the metastatistic lesions and accepted the immunotherapy of nivolumab. However, the tumor’s progression persisted and the patient succumbed to to the disease 7 months later after operation.

## Discussion

Mediastinum is one of the most common sites of HV-CD. In this perplexing case, we still can trace the characteristic profiles of intranodal HV-CD, e.g., the atretic follicles with proliferative dendritic cells, concentrically-arrayed lymphocytes in mantle zone, and expended interfollicular zone with abundant small vessels. However, it is challenge to explain the nature of those proliferated spindle cells, which demonstrated features varying from bland morphology to remarkable atypia.

Interestingly, Danon et al. (5) had described a florid proliferation of the interfollicular spindle cells in a few cases of HV-CD they enrolled. Those cells showed mild ovoid vesicular and plump nuclei. Their volume proportion was generally greater than 75% of the whole lesion and the follicles in between were atrophied. The spindle cells were focally immunoreactive with actin and CD68. The name of SR-HVCD was then proposed for such lesions. Subsequently, Lin et al. and Miki et al. (6, 7) respectively reported similar cases, characterized by the overgrowth of spindle cells in the interfollicular areas. Positive staining of SMA and CD68 were also found in a subset of those cells. Based on the morphologic and immunophenotypic features, the spindle cells were postulated to be derived from the fibroblastic, myofibroblastic, histiocytic cells, and/or vessel-related pericytes in the lymph node. So the angiomyoid proliferative lesion of HV-CD was alternatively used to describe such disorders (5), which particularly highlights the cellular composition inside the disorder. More importantly, all the reported cases exhibited a benign course and no recurrence was occurred after excision.

Our case was most likely conformed to the characteristics of SR-HVCD, whereas the interfollicular spindle cells exhibited remarkably atypia in some regions. Miki et al. specifically emphasized that their six cases of SR-HVCD were cytologically benign, lack of mitoses and necrosis. In addition, there were very low Ki-67 index and negative P53 expression (7). By contrast, the dysplastic spindle cells in our case demonstrated atypical mitoses and necrosis, and showed strong P53 expression and high Ki-67 index. Those characteristics obviously deviated from the profiles of previous reports and largely suggested it was a malignant process. The subsequent metastatic event also supported this speculation.

To clarify the nature of those interfollicular spindle cells, a substantial examination, including immunohistochemistry and FISH, was performed and showed the spindle cells, either in bland or atypical morphology, only displayed focal reaction with SMA and CD68. On the one hand, the results indicated, in consistence with earlier reports, they are probably originated from the fibroblastic, myofibroblastic, and/or histiocytic cells. On the other hand, a list of differential diagnosis was excluded: follicular dendritic cell sarcoma (CD21-, CD23-, CD35-, CXCL-13-, D2-40-, and SSTR2-), interdigitating dendritic cell sarcoma (S100-), indeterminate dendritic cell tumor (S100- and CD1a), Kaposi’s sarcoma (HHV8-, CD34-, CD31-, and ERG-), solitary fibrous tumor (CD34- and STAT6-), malignant peripheral nerve sheath tumors (S100-, H3K27me3+, and SOX10-), NTRK gene fusion-positive tumors (TRK-, CD34-, and S100-), melanoma (S100-, SOX10-, HMB45-, Melanoma A-, and p16-), inflammatory myofibroblastic tumor (CK- and ALK-), poorly-differentiated/undifferentiated carcinoma (CK-pan-, CK8/18-, EMA-, E-cadherin-, and EBER-), thymic carcinoma (CK-pan-, CK19-, CD5-, and CD117-), sclerosing epithelioid fibrosarcoma (EWSR1 gene rearrangement-, MUM4-), and synovial sarcoma (SS18 gene rearrangement-).

Notably, there are two intriguing issues that remain unsettled. First, if the pleomorphic spindle cells were considered as malignancy, what type of tumor should they be classified? Undifferentiated sarcoma (UC) might be a possibility based on the cells’ immunstaining features (SMA+ and CD68+). However, the atypical cellular component was generally self-limited inside the lesion, and no destruction or invasion to the adjacent structures was observed in our case. In addition, the nature of the metastatic lesion was regrettably kept unknown. So it seems insufficient to draw a diagnosis of UC based on the available evidences. Additional accumulated cases are expected to further clarify this question. Second, whether there existed a potential link between the atypical and bland stromal cells in pathogenesis. We assume the association could not be ruled out for the below reasons: (1) The two types of spindle cells were focally adjacent or intermingled inside the lesion, and a morphological transition could be perceived between them. (2) Both of them are supposed to be derived from fibroblastic, myofibroblastic, and/or histiocytic cells. Attractively, the acquired expression of p53 protein in the atypical spindle cells possibly indicated the p53 genetic alteration may trigger and/or contribute to their oncogenesis. In fact, p53 gene mutation has been demonstrared to play pivotal roles in the tumorigenesis of a variety of neoplasms (18, 19). A clonality analysis on the two cellular components might be helpful to fully unravel their internal relationship.

Finally, we also noticed the indolent T-lymphoblastic proliferation (iT-LBP) inside the lesion. Those T-lymphoblastic cells exhibited the phenotypes of precursor cortical thymocytes (TDT+, CD3+, CD5+, and CD10+), while presented no cytologic atypia. The TCR receptor rearrangement was not detected. Those features satisfied the diagnostic criteria of iT-LBP, as proposed by Ohgami et al. (20). It is worth noting that iT-LBP is frequently concurrent in disorders associated with the follicular dendritic cells’ proliferation, such as HV-CD, FDCS, and angioimmunoblastic T-cell lymphoma. The dysfunction of follicular dendritic cells may create an extrathymic immunomilieu benefiting for the development of iT-LBP (21).

In summary, we described a rare case of SR-HVCD that was featured by the atypical hyperplasia of the stromal cells and demonstrated a malignant behaviour. The atypical stromal cells acquired aberrant P53 protein expression. Although the etiology remains to be further clarified, it is likely the first report that manifests SR-HVCD has a tumorigenesis potential and challenges the conventional viewpoint that SR-HVCD is a simple benign and hyperplastic disorder. Acquaintance with this entity would favor pathologists and clinicians to make an inerrant diagnosis and treatment in practice.

## Data availability statement

The original contributions presented in the study are included in the article/**Supplementary Material**. Further inquiries can be directed to the corresponding author.

## Ethics statement

The studies involving human participants were reviewed and approved by Peking University Shenzhen Hospital. The patients/participants provided their written informed consent to participate in this study. Written informed consent was obtained from the individual(s) for the publication of any potentially identifiable images or data included in this article.

## Author contributions

XS, JL, and WY were engaged in the pathological diagnosis. XS, ML, and JL drafted the manuscript. XY, YC, and CH was involved in the immunohistochemical and molecular studies. All authors contributed to the article and approved the submitted version.

## Supplementary material

Supplementary Figure 1 Another field demonstrates the morphologic transition of the spindle cells from mild morphology (left side of the dot line) to marked atypia (right side of the dot line) **(A)**. In the former region (the red square), the spindle cells exhibit vesicular to fine chromatin, and inconspicuous small nucleoli **(B)**. In contrast, the cells in the latter region (the yellow square) illustrate enlarged, irregular nuclei and atypical mitosis (white arrow) **(C)**.

Supplementary Figure 2 The atypical spindle cells demonstrate the hemangiopericytoma-like arrangement (white arrow) and focal necrosis (black arrow).

Supplementary Figure 3 Hyperplastic small vessels are intermixed with the compact atypical spindle cells. They are not easily detectable on H&E section, while could be highlighted by CD34 staining.

Supplementary Figure 4 The atypical spindle cells show negative staining with CD21, CD23, CD35, SSTR2, D2-40, and CXCL13.

Supplementary Figure 5 Magnetic resonance imaging shows nodular abnormal signals (white arrow) in the liver, right paraspinal **(A)** and vertebral bodies **(B)**.
