# Supplementary figures and images for: Case report: The stroma-rich variant of Castleman’s disease of hyaline-vascular type with atypical stromal cell proliferation and malignant potential: An exceptional rare case occurred in mediastinal lymph node
Source: Front Oncol. 2023 Mar 16;13:1008587. doi: 10.3389/fonc.2023.1008587 (PMC10061130; doi:10.3389/fonc.2023.1008587)

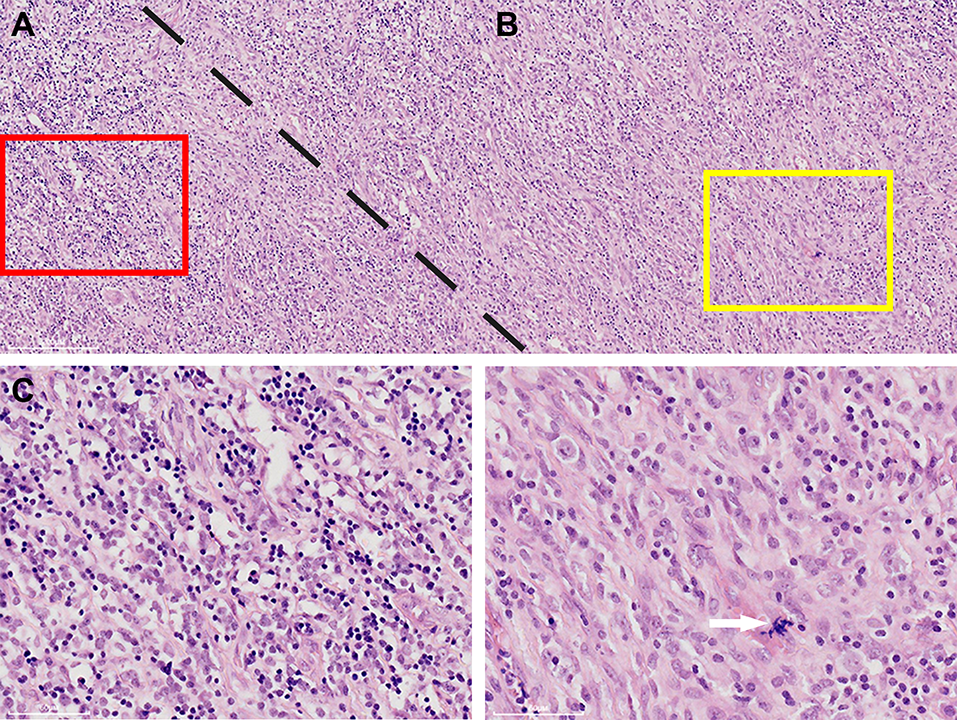

Supplement: Supplementary Figure 1 — Another field demonstrates the morphologic transition of the spindle cells from mild morphology (left side of the dot line) to marked atypia (right side of the dot line) (A). In the former region (the red square), the spindle cells exhibit vesicular to fine chromatin, and inconspicuous small nucleoli (B). In contrast, the cells in the latter region (the yellow square) illustrate enlarged, irregular nuclei and atypical mitosis (white arrow) (C). [file Image_1.tif]

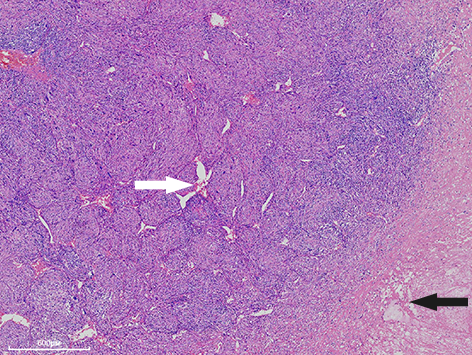

Supplement: Supplementary Figure 2 — The atypical spindle cells demonstrate the hemangiopericytoma-like arrangement (white arrow) and focal necrosis (black arrow). [file Image_2.tif]

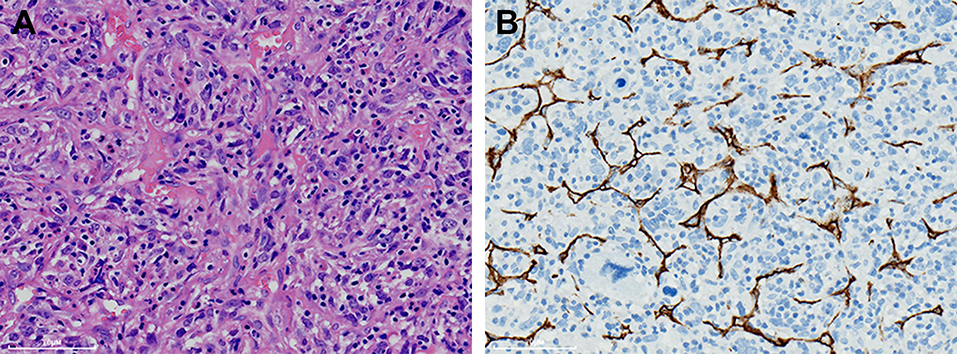

Supplement: Supplementary Figure 3 — Hyperplastic small vessels are intermixed with the compact atypical spindle cells. They are not easily detectable on H&E section, while could be highlighted by CD34 staining. [file Image_3.tif]

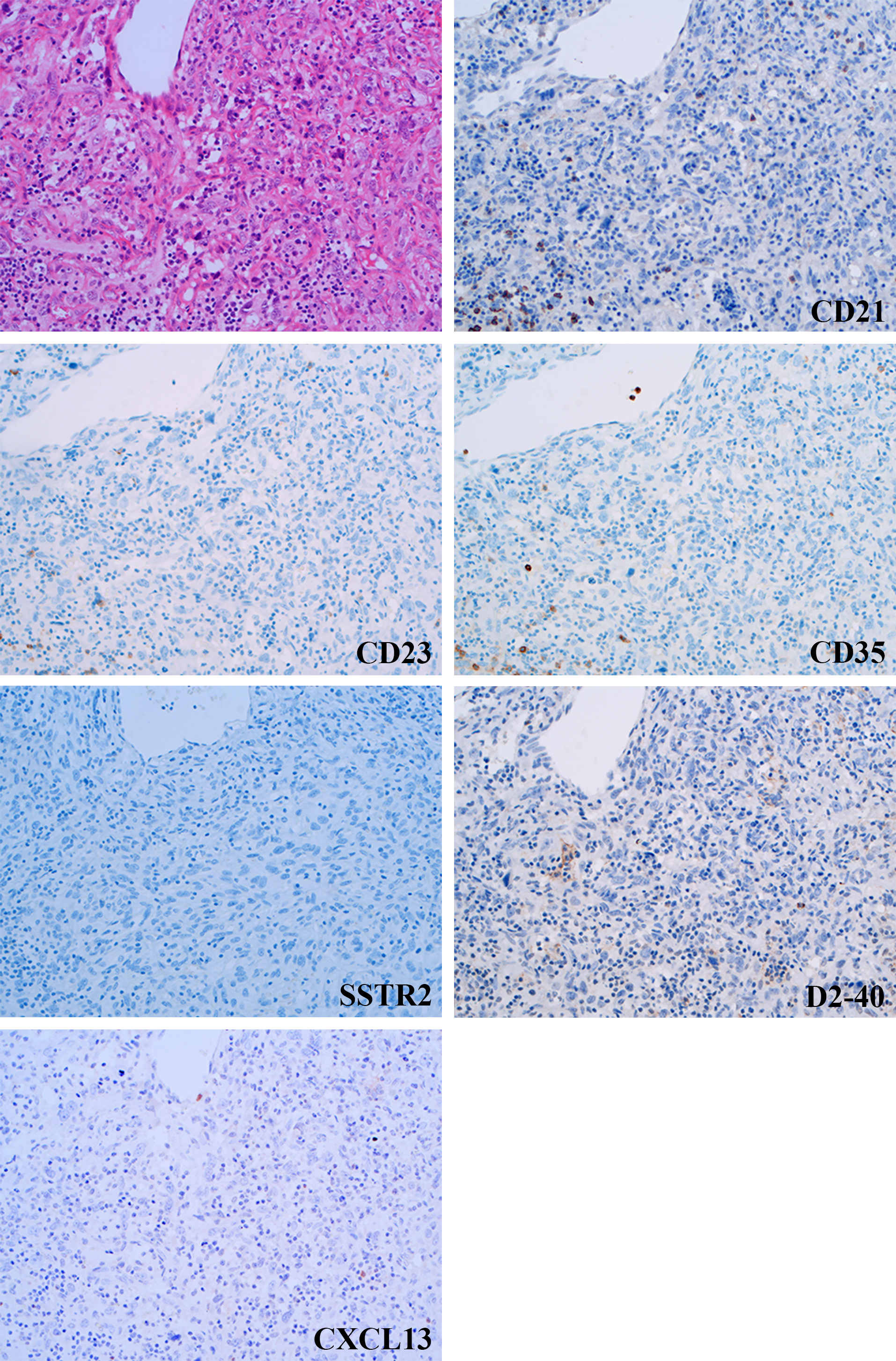

Supplement: Supplementary Figure 4 — The atypical spindle cells show negative staining with CD21, CD23, CD35, SSTR2, D2-40, and CXCL13. [file Image_4.tif]

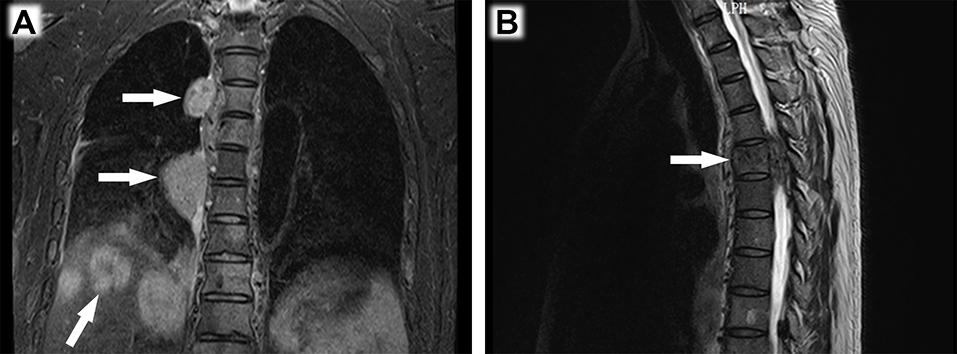

Supplement: Supplementary Figure 5 — Magnetic resonance imaging shows nodular abnormal signals (white arrow) in the liver, right paraspinal (A) and vertebral bodies (B). [file Image_5.tif]
